# Sex Differences in Epicardial Adipose Tissue and Other Risk Factors for Coronary Artery Disease

**DOI:** 10.3390/medicina61050934

**Published:** 2025-05-21

**Authors:** Vesna Lesjak, Laura Kocet

**Affiliations:** 1 Radiology Department, University Medical Centre Maribor, 2000 Maribor, Slovenia; laura.kocet@ukc-mb.si; 2 Faculty of Health Sciences, University of Ljubljana, 1000 Ljubljana, Slovenia

**Keywords:** epicardial adipose tissue attenuation, coronary artery disease, sex differences, coronary artery calcification, risk factors

## Abstract

*Background and Objectives:* To examine individual-level sex differences in traditional and non-traditional risk factors and their potential effects on the severity of coronary artery disease (CAD). *Materials and Methods:* A cross-sectional analysis was performed on 208 patients with a low-to-intermediate pretest probability of CAD, referred to a Coronary CT angiography (CCTA) at the Department of Radiology, Maribor University Medical Centre, from January 2022 to January 2024. CCTA-derived EAT (epicardial adipose tissue) attenuation and CAC (coronary artery calcification) values were measured. The association between CAD, EAT, and risk factors was analyzed by sex, using correlation analysis and multivariate regression. *Results:* In the results obtained using the univariate logistic regression model, age (OR 1.122, *p* < 0.001) and hypertension (OR 4.087, *p* = 0.048) were significantly associated with the presence of obstructive CAD in women, while in men, age (OR 1.052, *p* = 0.008), hypercholesterolemia (OR 3.765, *p* = 0.042), and EAT attenuation (OR 1.053, *p* = 0.011) were significant factors. In results obtained using the multivariable logistic regression analysis model, EAT attenuation was found to be significantly associated with the presence of obstructive CAD in men (OR 1.087, *p* = 0.012), and age was a significant factor in women (OR =1.108, *p* = 0.033), while hypertension, body mass index (BMI), diabetes, hypercholesterolemia, angina pectoris, and smoking were not. *Conclusions:* In the sex-specific multivariable logistic regression analysis model, EAT attenuation was significantly associated with obstructive CAD in men, while in women, it was associated with age. EAT may function as a beneficial alternative indicator in identifying patients with CAD.

## 1. Introduction

Worldwide, cardiovascular diseases are the main cause of morbidity and mortality. Ischemic heart disease is the leading cause of mortality in the world, with 9.0 million deaths attributed to it in 2021 [1]. Even though women often exhibit a lower prevalence of cardiovascular disease compared to men, several clinical studies showed that women have a higher mortality rate and worse prognosis after an acute event [2]. The misperception that women are more protected against cardiovascular disease than men frequently results in the underestimation of the threat of cardiovascular complaints in women. Women are less likely to be referred for ischemia functional testing, and women’s treatment strategies are often less aggressive compared to men’s treatment [3,4].

Studies indicate that women with coronary artery disease (CAD) are often older than their male counterparts, have a greater prevalence of cardiovascular risk factors [5], and are more likely to have several comorbidities present. Traditional risk factors include dyslipidemia, hypertension, diabetes, obesity, and tobacco use [6]. However, up to one-fifth of individuals experiencing myocardial infarction lack traditional risk factors [7]. Non-traditional risk factors for CVD (cardiovascular disease) include reproductive factors, chronic inflammation (women more often have autoimmune diseases), socioeconomic status, as well as depression, sleep duration, and emotional and household-related stress [6,8].

Calcifications are a component of the atherosclerotic process. They are associated with the same risk factors and are primarily located in advanced lesions [9]. Studies indicate an association between the absence of coronary artery calcification (CAC) and a very low risk of future cardiovascular events [10]. However, a lack of CAC cannot undoubtedly exclude stenosis of a coronary artery, and a high CAC does not necessarily correlate with coronary artery stenosis [11]. There are also differences in coronary artery calcification; in general, the prevalence and extent of coronary plaque burden are higher in men than women [12]. Women exhibit a higher propensity for plaque erosion without concurrent CAC. This trait is significant as it affects the diagnostic procedure—smaller arteries and a reduced necrotic core are prevalent, making the coronary artery calcium score (CACS) less indicative of cardiovascular events than in men [13].

Epicardial fat is a visceral adipose tissue surrounding the myocardium and coronary arteries within the pericardium [14]. The correlation between epicardial adipose tissue attenuation, coronary artery plaque burden, and coronary artery disease is well-documented in the literature [15].

The lipid content and size of adipocytes can be described by the density of adipose tissue, which indirectly reflects the inflammation and fibrosis of the tissue. An adverse metabolic profile was documented to be associated with a lower EAT density in the general population and patients with a higher risk for CVD. Also, the prognosis of an asymptomatic individual was susceptible to a reduction in EAT density [16].

A comprehensive study of the sex differences in the risk factors associated with CAD could result in better CVD outcomes, especially in women. Therefore, our study aimed to examine individual-level sex differences in the CT-derived EAT attenuation, traditional and non-traditional risk factors, and their potential effects on the severity of coronary artery disease.

## 2. Materials and Methods

We performed a cross-sectional study and included all patients with a low-to-intermediate pretest probability of CAD, referred to a Coronary CT angiography (CCTA) at the Department of Radiology, Maribor University Medical Centre, from 7 January 2022 to 8 January 2024. Patients with a prior history of a CABG (coronary artery bypass graft), coronary stent, or cancer were excluded, as well as those who had a baseline estimated glomerular filtration rate (eGFR) of less than 30 mL/min per 1.73 square metres (m^2^) or had poor imaging quality. After providing written informed consent, participants responded to a questionnaire about demographic data and clinical history. Baseline demographics and medical history were obtained. Structured questionnaires were used for self-reported data about weight and height, smoking, presence of hypertension, hypercholesterolemia, and diabetes mellitus. Subjects were asked: “How many hours of sleep do you usually get each night?”. The reported sleep duration was categorized into three categories for data analysis, in accordance with previous studies [17]: <6 h per day, ≥6 to <9 h per day, or ≥9 h per day.

Cardiac CT scans were performed on a Somatom Drive (Siemens Medical Solutions, Erlangen, Germany) CT scanner. Quantification of coronary artery calcification was performed on a non-contrast scan using the Agatson method, with the use of software for semi-automated assessment of calcium in the coronary arteries (Syngo via VB10, Siemens Healthcare, Forchheim, Germany), as previously detailed [18]. Epicardial fat attenuation was measured on non-contrast axial images. We traced the region of interest (ROI) manually to measure the attenuation of epicardial adipose tissue HUs (Hounsfield units) on the axial slices, near the proximal part of the right coronary artery (RCA). The ROI was delineated in a single slice, at a location that is not contiguous with CAC and is free from streak or motion artefacts, as previously described [19]. The same experienced investigator (L.V.) performed all epicardial adipose tissue attenuation measurements.

To determine coronary artery disease, imaging was performed during a single breath hold in the craniocaudal direction, with retrospective ECG gating. Patients with a heart rate over 80 beats per minute received beta-blockers to minimize artefacts. All image analyses were performed on a dedicated workstation, and coronary arteries were evaluated for obstructive coronary disease. Obstructive CAD was defined as an epicardial vessel with a stenosis of ≥50 percent.

The data collected were analyzed using SPSS version 29.0 for Windows (IBM Inc., Armonk, NY, USA). Statistical significance was set at a *p*-value less than 0.05. Mann–Whitney U test was employed to compare continuous variables between groups, and they were given as median and interquartile range (IQR) as mean ± standard deviation (SD). Categorical variables were compared using the chi-square or Fisher’s exact test, as appropriate, and given as frequencies and percentages. Pearson or Spearman rank correlations were calculated to determine the relationships between EAT attenuation and continuous risk factors. Univariate and multivariate analysis was performed for independent associations of CAD, using stepwise multivariable logistic regression models with forward stepwise elimination, based on criteria of *p* ≤ 0.10 to enter and *p* ≤ 0.05 to stay in the model. The prevalence of obstructive CAD was compared according to the number of risk factors present (smoking, hypertension, hypercholesterolemia, diabetes, and BMI > 30). To provide a potentially clinically useful threshold for EAT attenuation and CACS, optimal cut-off values regarding obstructive CAD were calculated with receiver operating characteristic (ROC) curve analysis.

This study meets the guidelines of the Declaration of Helsinki and was approved by the local ethics committee.

## 3. Results

The demographic characteristics of participants are shown in Table 1. In total, 50.5% of the 208 participants were men. Mean ± SD age was 62.3 ± 11.1 years in women and 57.2 ± 12.0 years in men (*p* = 0.001). Significant gender differences were found regarding the presence of angina pectoris, obstructive coronary artery disease, and coronary artery calcifications. Angina pectoris was more prevalent among women (52.9% of women vs. 36.9% of men, *p* = 0.015). Women were more likely to have a calcium score of zero than men (*p* = 0.040). Men were more likely to have obstructive CAD and higher coronary artery calcification than women. Mean EAT attenuation was lower in women than men (−105.3 ± 13.8 HU vs. −98.4 ± 11.2 HU, *p* < 0.001).

Significant sex differences were found regarding the presence of hypertension, hypercholesterolemia, smoking, elderly patients, EAT attenuation, and hours of sleep comparing the groups with and without obstructive CAD (Table 2). Women with obstructive CAD were significantly older and had more hypertension, smoked more, had higher CACS, and slept fewer hours per night than those without obstructive CAD (*p* < 0.001, *p* = 0.013, *p* = 0.031, *p* < 0.001 and 0.008, respectively). Men with obstructive CAD were older, had more hypercholesterolemia, higher EAT attenuation, and higher CACS than those without obstructive CAD (*p* = 0.006, *p* = 0.037, *p* = 0.006, and *p* < 0.001).

The univariate analysis showed that age (OR 1.058, *p* < 0.001), male gender (OR 2.140, *p* = 0.014), hypertension (OR 2.359, *p* = 0.008), hypercholesterolemia (OR 1.942, *p* = 0.046) and EAT attenuation (OR 1.032, *p* = 0.013) are predictors that were significantly associated with the presence of obstructive CAD (Table 3). Age (OR 1.122, *p* < 0.001) and hypertension (OR 4.087, *p* = 0.048) were significantly associated with the presence of obstructive CAD in women, while in men, predictors that were significantly associated with the presence of obstructive CAD were age (OR 1.052, *p* = 0.008), hypercholesterolemia (OR 3.765, *p* = 0.042), and EAT attenuation (OR 1.053, *p* = 0.01).

In the multivariable logistic regression analysis model, EAT attenuation and hypertension were significantly associated with obstructive CAD (OR 1.049, *p* = 0.014 and OR 3.377, *p* = 0.020). In the sex-specific multivariable logistic regression analysis model, EAT attenuation was found to be significantly associated with the presence of obstructive CAD in men (OR 1.087, *p* = 0.012). In women, age was found to be significantly associated with the presence of obstructive CAD (OR =1.108, *p* = 0.033), while hypertension (OR = 7.560, *p* = 0.075), BMI, diabetes, hypercholesterolemia, angina pectoris, and smoking were not.

**Figure 1:**
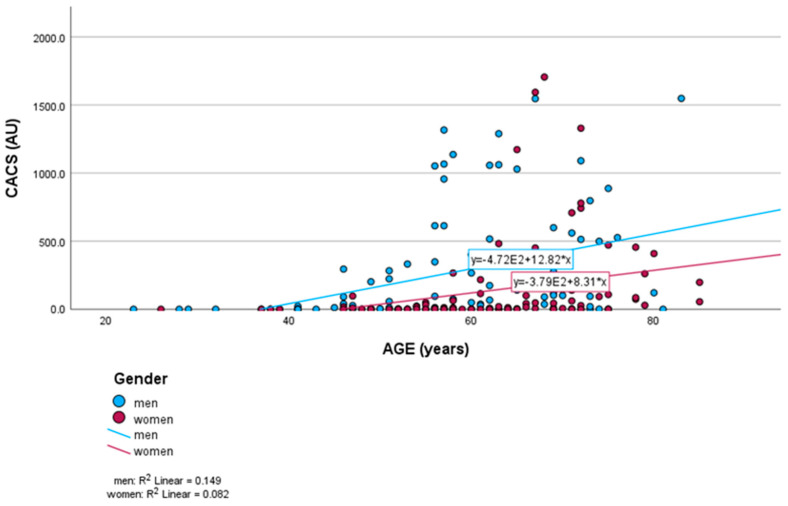
Association between age and CACS in women and men. CACS: coronary artery calcium score.

A total of 33.7% of participants had a calcium score of zero, while 20.2% had a CACS over 400. In total, 4.3% of women with obstructive CAD had no coronary artery calcifications, compared to none of the men, while CACS > 400 was present in 47.8% of women and 57.5% of men (*p* < 0.001). Men had higher CACS values than women. Also, men had higher CACS values at a younger age than women (Figure 1).

**Figure 2:**
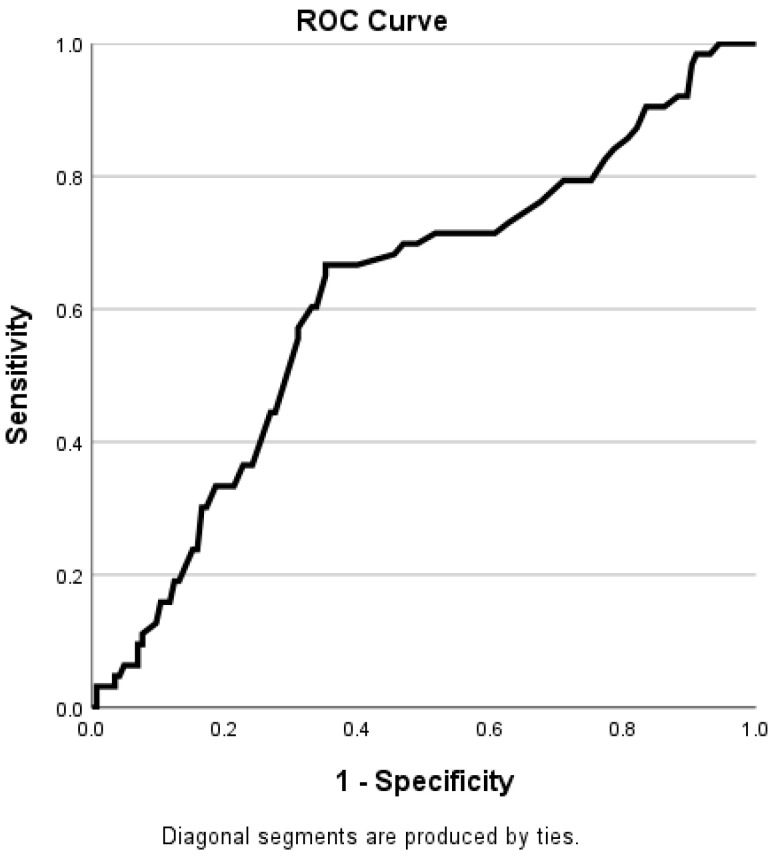
The receiver operating curve (ROC) analysis of EAT attenuation value discriminates between patients with obstructive CAD and those without obstructive CAD. AUC = 0.618 (95% CI = 0.534–0.702, *p* = 0.006).

The ROC curve analysis (Figure 2) showed that the optimal cut-off for EAT attenuation value to discriminate between obstructive and non-obstructive CAD was 98.750, with a sensitivity of 66.7% and specificity of 64.8%, with an area under the curve (AUC = 0.618; 95% confidence interval [CI] = 0.534–0.702, *p* = 0.006). The Youden J statistic was found to be 0.315 for the cut-off level. No statistically significant differences existed between men and women in the ROC curve analysis.

## 4. Discussion

Traditional factors for coronary artery disease include age, sex, diabetes, hypertension, dyslipidemia, and smoking in both sexes [20]. Obstructive coronary disease, defined as the presence of at least one coronary artery stenosis > 50%, is more common in men, and our study confirms this (38.8% of men versus 22.1% of women). CAD significantly correlates with age, with older age being a common risk factor in both sexes, which is consistent with our results. In our study population, females with obstructive CAD were older than males (70.5 ± 7.1 years vs. 61.3 ± 8.8 years). Estrogen is believed to delay the manifestation of atherosclerosis in women before menopause [21]. Post-menopause, estrogen deficiency results in a significant escalation of cardiovascular risk due to its induction of various structural and functional alterations in the cardiovascular system, including endothelial dysfunction, an imbalance in autonomic regulation favouring increased adrenergic activity, visceral obesity, and systemic inflammation [4]. Persistent low-grade inflammation, characterized by moderate increases in circulating pro-inflammatory cytokines (C-reactive protein: CRP; interleukins (IL): IL-6, IL-2, IL-7, IL-8, etc.), plays a significant role in all atherosclerosis formation and progression phases. The perception of gender-specific protection of estrogen results in diminished aggressiveness and delayed preventative and/or treatment methods, thereby exacerbating women’s coronary artery disease [8].

Women frequently exhibit multi-vessel disease and less obstructive plaque, which has been ascribed to smaller coronary vessel anatomy, relatively low flow reserve, and increased arterial stiffness, compared to men [22]. Men typically exhibit a higher prevalence of calcified and fibrous plaques [23], while women are more frequently characterized by mixed or fibro-fatty plaques. This study also demonstrated that women more often had no calcification in coronary arteries than men and that men have a higher prevalence of calcified plaques. In women, acute events are more often attributed to plaque erosion than to plaque rupture, compared to men [24]. This characteristic is significant as it affects the diagnostic process; in women, smaller blood vessels and a reduced necrotic core are prevalent, making the CACS less predictive of cardiovascular events than in men. Estrogen significantly influences these sex differences, especially through its protective effect on coronary artery calcification [20,25,26]. Consequently, elevated CAC is infrequently observed in younger women but becomes prevalent with older age.

In this study, EAT attenuation was significantly higher in men than in women. The difference could be clinically meaningful. Men with obstructive CAD had significantly higher EAT attenuation than those without obstructive disease, while in women, the difference was present, but not significant. In the sex-specific multivariable logistic regression analysis model, EAT attenuation was significantly associated with obstructive CAD in men, but not in women. Increased EAT attenuation may indicate inflammation within the epicardial fat [27], and through its paracrine effects or systemic processes, contribute to the development of coronary atherosclerosis [28], which could contribute to the higher prevalence of calcified plaques in men. More studies should correlate EAT attenuation with local and systemic processes for further insight into the underlying processes.

Although the ROC analysis identified −98.75 HU as the potential threshold of EAT attenuation value to discriminate between obstructive and non-obstructive CAD, the associated AUC of 0.618 indicates only modest discriminatory ability. Therefore, the clinical utility of this threshold should be interpreted with caution. Validation in independent cohorts and supplementary tools such as decision curve analysis may help determine whether this threshold offers any net clinical benefit beyond existing diagnostic criteria. Ultimately, this value should not be used in isolation but as part of a comprehensive diagnostic framework.

Studies have shown that women with symptoms of CAD are more likely to have hypertension, hypercholesterolemia, diabetes, and angina pectoris than males [5,26,29]. They are also less likely to have had a myocardial infarction in the past or smoke. Our study confirms these results. The prevalence of hypertension is elevated in women aged above 60 years. Women are less inclined to obtain medical therapy for hypertension and exhibit worse blood pressure regulation [21]. Midlife hypertension increases the risk of myocardial infarction and is more detrimental to women than to men of the same age [30]. In our study, in the sex-specific multivariable logistic regression analysis model, hypertension was not found to be significantly associated with the presence of obstructive CAD (*p* = 0.075, OR = 7.560), which could be due to a small sample size or possible underpowering.

Sleep is a vital physiological function that safeguards both physical and mental well-being. Sleep deprivation is ubiquitous in the Western world, and epidemiological research indicates that both short and prolonged sleep durations are associated with heightened cardiovascular risk [31]. Studies have reported an association between short sleep duration, hypertension [32], obesity, and food intake [33]. Also, sleep duration and quality have been associated with diabetes mellitus, stroke, coronary heart disease, and subclinical atherosclerosis [34]. A U-shaped association was observed between self-reported sleep duration and the degree of coronary artery calcification in a cross-sectional study of apparently healthy women and men [35]. We found an association between sleep duration and obstructive CAD in women, not men. There is no known gender effect on sleep efficiency or length [17]; however, gender-related variables such as household chores and caring for children might adversely affect outcomes in women relative to males [8].

Although biological factors may be more advantageous, gender-related variables adversely affect outcomes in females with coronary artery disease relative to males [8]. Females are more susceptible to health disparities that are influenced by political and socioeconomic circumstances, as well as social and cultural factors, than their male counterparts [36].

When interpreting the results of this study, some limitations must be considered. First, the sample size is quite small, although there is an equal representation of women and men. Our study was cross-sectional; therefore, it cannot prove causality, and caution is needed with etiological conclusions. An additional limitation could be that the presence of chronic diseases, sleep duration, and weight and height were self-reported. This could induce misclassification bias in our adjusted associations. Ultimately, residual confounding may arise from unassessed variables.

The biological distinctions and underlying sex-specific pathophysiology of cardiovascular disease in women remain inadequately understood. A comprehensive approach is necessary to elucidate the mechanisms of coronary artery disease in men and women, including sex differences, including genetic, inflammatory, metabolic, and environmental variables. Future research must concentrate on inequalities, clarifying the women-specific pathways and risk factors with prospective studies and clinical trials, utilizing a blend of genetic, molecular, imaging, and clinical strategies, to improve our understanding of the fundamental processes.

## 5. Conclusions

This study demonstrates that the EAT attenuation was significantly associated with obstructive CAD in men in the sex-specific multivariable logistic regression analysis model, but not in women, while other risk factors were not. Care is needed with the etiological implications of our study, and the EAT attenuation should not be used in isolation but rather as part of a comprehensive diagnostic framework. However, we believe that EAT may be a beneficial alternative indicator in identifying patients with CAD, as it may reflect the variations in the composition and/or function of epicardial fat. However, coronary artery disease is a multifactorial disorder, arising from a complex interplay of several genetic, environmental, and lifestyle factors, the complexity of which remains a research focus for future studies.

## Figures and Tables

**Table 1:** Comparison of parameters (demographic, clinical parameters, and CT-derived variables) between sexes.

Variable	All (n = 208)	Men (n = 105)	Women (n = 103)	*p*-Value
BMI (kg/m^2^)	29.2 ±5.6	29.5 ± 5.1	28.9 ± 6.2	0.334
Age (years)	59.7 ± 11.8	57.2 ± 12.0	62.3 ± 11.1	0.001
EAT attenuation	−101.8 ± 12.9	−98.4 ± 11.2	−105.3 ± 13.8	<0.001
CACS (AU)	344.6 (268.0)	484.1 (515.2)	202.3 (121.3)	0.003
CACS(AU) = 0	70 (33.7%)	28 (26.7%)	42 (40.8%)	0.040
CACS per vessel				
LMA	45.9 (34.8)	51.9 (51.3)	39.8 (21.8)	0.077
LAD	122.7 (92.9)	117.9 (191.2)	66.4 (59.3)	0.009
LCX	50.5 (13.3)	73.4 (29.5)	27.2 (6.2)	0.004
RCA	124.1 (28.8)	177.3 (63.5)	69.9 (3.9)	0.001
Smoking	37 (17.9%)	23 (22.3%)	14 (13.4%)	0.110
Diabetes	30 (14.5%)	15 (14.6%)	15 (14.4%)	0.997
Hypertension	114 (55.1%)	52 (50.4%)	62 (59.6%)	0.140
Hypercholesterolemia	54 (26.1%)	21 (20.4%)	33 (31.7%)	0.052
Family history of cardiovascular disease	122 (58.9%)	59 (57.3%)	63 (60.5%)	0.517
Myocardial infarction	11 (5.3%)	7 (6.8%)	4 (3.8%)	0.361
Angina pectoris	93 (44.9%)	38 (36.9%)	55 (52.9%)	0.015
Presence of at least one coronary artery stenosis ≥ 50%	63 (30.3%)	40 (38.8%)	23 (22.1%)	0.013
Presence of at least one coronary artery stenosis ≥ 70%	27 (13.0%)	19 (18.4%)	8 (7.7%)	0.027

Values are presented as mean ± SD (standard deviation), median (IQR, interquartile range), or n (%, percentages). LMA: left main coronary artery; LAD: left anterior descending artery; LCX: left circumflex artery; RCA: right coronary artery.

**Table 2:** Clinical characteristics of the study subjects with and without obstructive coronary artery disease (CAD).

	Men		*p*-Value	Women		*p*-Value
	CAD > 50%	CAD < 50%		CAD > 50%	CAD < 50%	
BMI	30.28 ± 4.57	29.1 ± 5.3	0.217	29.2 ± 6.5	28.8 ± 6.1	0.798
Age	61.3 ± 8.8	54.9 ± 12.9	0.006	70.5 ± 7.1	60.3 ± 10.9	<0.001
Age > 65 years	13 (32.5%)	14 (21.5%)	0.212	18 (78.3%)	25 (31.3%)	<0.001
EAT attenuation	−94.9 ± 10.8	−100.5 ± 10.9	0.006	−104.3 ± 12.8	−105.5 ±14.1	0.797
CACS (AU)	1041.6 (854.3)	142.5 (66.5)	<0.001	604.6 (828.8)	85.8 (39.8)	<0.001
CACS (AU) = 0	0 (0%)	28 (43.1%)	<0.001	1 (4.3%)	41 (51.2%)	<0.001
CACS per vessel						
LMA	93.7 (114.0)	26.3 (5.0)	<0.001	117.1 (117.8)	17.5 (0.0)	<0.001
LAD	391.7 (419.5)	46.8(18.8)	<0.001	186.0 (342.0)	31.7 (11.0)	<0.001
LCX	180.1 (183.0)	7.9 (2.7)	<0.001	88.8 (71.5)	9.3 (0.0)	<0.001
RCA	375.5 (341.0)	55.8 (1.0)	<0.001	217.3 (159.9)	27.3 (0.0)	<0.001
Smoking	12 (30.7%)	11 (16.9%)	0.100	0	14 (17.5%)	0.031
Diabetes	5 (12.8%)	10 (15.4%)	0.719	4 (17.4%)	11 (13.7%)	0.663
Hypertension	24 (61.5%)	28 (43.1%)	0.068	19 (82.6%)	43 (53.7%)	0.013
Hypercholesterolemia	12 (30.8%)	9 (13.8%)	0.037	10 (43.5%)	23 (28.7%)	0.182
Family history of cardiovascular disease	22 (56.4%)	37 (56.9%)	0.959	12 (52.2%)	51 (63.7%)	0.315
Myocardial infarction	4 (10.3%)	3 (4.6%)	0.266	2 (8.7%)	2 (2.5%)	0.175
Angina pectoris	17 (43.6%)	21 (32.3%)	0.247	12 (52.2%)	43 (53.7%)	0.894
Hours of sleep			0.733			0.008
<6	7 (31.8%)	11 (26.1%)		4 (40%)	17 (37.8%)	
6–9	14 (63.6%)	27 (64.3%)		4 (40%)	28 (62.2%)	
>9	1 (4.6%)	4 (9.5%)		2 (20%)	0	

Values are presented as mean ± SD (standard deviation), median (IQR, interquartile range), or n (%, percentages).

**Table 3:** Results of univariate logistic regression analysis with CAD > 50% as the dependent factor.

Variable		All		Male		Female	
	Units	OR	*p*-Value	OR	*p*-Value	OR	*p*-Value
Age	+1 year	1.058	<0.001	1.052	0.008	1.122	<0.001
Gender	Male vs. female	2.140	0.014				
BMI	+1 kg/m^2^	1.030	0.227	1.048	0.258	1.010	0.795
Diabetes	Present vs. absent	1.003	0.995	0.719	0.809	1.321	0.663
Hypertension	Present vs. absent	2.359	0.008	2.114	0.07	4.087	0.048
Hypercholesterolemia	Present vs. absent	1.942	0.046	2.765	0.042	1.906	0.186
Smoking	Present vs. absent	1.152	0.716	2.182	0.104	0.000	0.999
EAT	+1 HU	1.032	0.013	1.053	0.011	1.005	0.068

OR: odds ratio; BMI: body mass index; EAT: epicardial adipose tissue attenuation.

## Data Availability

Data are available upon reasonable request from the corresponding author.

## Abbreviations

The following abbreviations are used in this manuscript:

EAT	Epicardial adipose tissue;
CAD	Coronary artery disease;
CCTA	Coronary computer tomography angiography;
CAC	Coronary artery calcifications;
BMI	Body mass index;
CVD	Cardiovascular disease;
CACS	Coronary artery calcium score;
CABG	Coronary artery bypass graft;
ROI	Region of interest;
HU	Hounsfield units;
LAD	Left anterior descending artery;
LCX	Left circumflex artery;
LMA	Left main coronary artery;
RCA	Right coronary artery.
